# PP77 Cost-Utility Analysis Of Nivolumab For Recurrent Or Metastatic Head And Neck Squamous Cell Carcinoma In The Brazilian Public Healthcare System

**DOI:** 10.1017/S026646232510295X

**Published:** 2025-12-29

**Authors:** Ludmila Gargano, Layssa Oliveira, Haliton Oliveira Junior, Rosa Camila Lucchetta

## Abstract

**Introduction:**

Head and neck squamous cell carcinoma (HNSCC) is the most common malignancy of the oral cavity, pharynx, and larynx. Nivolumab has demonstrated significant survival benefits in patients with recurrent or metastatic HNSCC after platinum-based chemotherapy. This study evaluated the cost utility of nivolumab compared with standard chemotherapy for recurrent or metastatic HNSCC in adults in the Brazilian Unified Health System (SUS).

**Methods:**

A partitioned survival model was developed using survival data from the CheckMate-141 trial. Health states included progression-free survival (PFS), post-progression survival, and death. Estimates for overall survival (OS) and PFS were extrapolated from Kaplan-Meier curves using parametric distributions. Costs and utilities were analyzed from the SUS perspective over a five-year horizon, with a five percent annual discount rate for both costs and benefits. Utilities were derived from international literature. Costs were measured in BRL and converted to purchasing-power parity in 2023 (USD1=BRL2.44). Incremental cost-utility ratios (ICUR) were calculated using quality-adjusted life years (QALYs) as the outcome.

**Results:**

Nivolumab resulted in incremental costs of USD56,040 (BRL136,737) and incremental benefits of 0.12 QALYs and 0.37 life years gained (LY), compared with chemotherapy, leading to an ICUR of USD458,039 (BRL1,117,615) per QALY gained, or USD152,588 (BRL372,315) per LY. This exceeded the commonly accepted threshold of USD49,180 (BRL120,000) per QALY for the SUS. Sensitivity analyses identified drug costs and utility values as the most impactful parameters. Despite benefits for OS (hazard ratio 0.68, 95% confidence interval [CI]: 0.54, 0.86), nivolumab was not cost effective under current thresholds due to its high costs and lack of effect on PFS (hazard ratio 0.89, 95% CI: 0.70, 1.13).

**Conclusions:**

While nivolumab offered significant clinical benefits, including improved OS and a favorable safety profile, its high incremental cost means it is not a cost-effective option for treating recurrent or metastatic HNSCC from the SUS perspective. Strategic pricing adjustments or alternative access mechanisms may be necessary to enable broader access to this drug in the Brazilian healthcare system.

